# Editorial: Microorganisms in dehalogenation: regulation and enhancement

**DOI:** 10.3389/fmicb.2025.1559619

**Published:** 2025-01-28

**Authors:** Guofang Xu, Honghong Lyu, Jingchun Tang

**Affiliations:** ^1^Department of Civil and Environmental Engineering, National University of Singapore, Singapore, Singapore; ^2^Tianjin Key Laboratory of Clean Energy and Pollution Control, School of Energy and Environmental Engineering, Hebei University of Technology, Tianjin, China; ^3^MOE Key Laboratory of Pollution Processes and Environmental Criteria/Tianjin Engineering Research Center of Environmental Diagnosis and Contamination Remediation, College of Environmental Science and Engineering, Nankai University, Tianjin, China

**Keywords:** dehalogenation, microorganisms, regulation, enhancement, microbial reductive

## Introduction

The extensive industrial and agricultural use of halogenated organic compounds poses significant environmental and public health challenges. These compounds, characterized by their toxicity, hydrophobicity, and resistance to degradation, accumulate in soil and groundwater, leading to long-term pollution (Ackerman Grunfeld et al., 2024; He et al., 2021). Organohalide-respiring bacteria (OHRB), including *Dehalococcoides, Dehalogenimonas*, and *Dehalobacter*, play a pivotal role in the transformation of these pollutants across diverse environments (Matturro et al., 2017; Qiu et al., 2020; Xu et al., 2024). However, microbial degradation of halogenated organic pollutants is sometimes inefficient. The degradation rates are typically low, and in some cases, these microbial transformation produces more toxic byproducts (Ding et al., 2013).

To address these challenges, innovative strategies are needed to regulate and enhance the metabolic activity of OHRB, accelerating the degradation of halogenated organic pollutants. This Research Topic presents a curated collection of cutting-edge studies, offering insights into microbial dehalogenation processes, interactions with functional materials, and integrated approaches for environmental remediation. By bringing together the six latest studies in this field, we hope to advance the understanding and application of integrated approaches for more efficient degradation and remediation of organohalide pollutants.

## Reflections on OHRB research trajectory

Liao et al. provided a timely bibliometric analysis of OHRB research over the past four decades, highlighting the remarkable expansion of this field and extensive collaboration among researchers. Their study identified two primary research foci, namely mechanistic exploration of OHRB and their interplay with the environment. Looking ahead, they outlined several priorities for advancing OHRB research, including the exploration of OHRB's role in biogeochemical cycles, the enhancement of their capabilities through synthetic biology, the investigation of their ecological interactions and evolutionary pathways, as well as the potential of other microorganisms (e.g., archaea) to perform dehalogenation.

## Cobamides: a key to unlocking OHRB potential

Lu et al. explored the critical roles of cobamides, an essential co-factor of reductive dehalogenases (RDases), in microbial reductive dehalogenation. Specifically, their review detailed the structural diversity of cobamides, their influence on dehalogenation activity, as well as the underlying catalytic mechanisms in organohalide respiration. Additionally, it underscored the importance of interspecies interactions between cobamides-producing microorganisms and OHRB. The cobamide producers supply critical cofactors that OHRB modify and assemble into functional RDases, highlighting the need to consider these cobamides-producers when designing robust dehalogenating consortia.

## Innovations in electron transfer mechanisms

Electron transfer processes are fundamental to microbial reductive dehalogenation. Eberwein et al. discovered that it is feasible to use cobalt-containing metal complexes (cobalt chelates) as anode mediators to replace halogenated compounds for the cultivation of *Dehalococcoides mccartyi* CBDB1 in bioelectrochemical systems. The cobalt chelates can putatively shuttle electrons between the organohalide respiration complex and the anode, coupling *D. mccartyi* CBDB1 cells to the electrode via mediated extracellular electron transfer. This provides a promising alternative to the traditional methods for the cultivation of OHRB, eliminating its reliance on toxic organohalides.

Humic compounds are well known for their ability to mediate electron transfer in diverse microbial processes, such as reduction of metal oxide and nitrate (Yang et al., 2021). Wang et al. investigated how humin, humic acid, and anthraquinone-2,6-disulfonic acid affect microbial reductive dechlorination of polychlorinated biphenyls (PCBs). Their study showed that the solid-state humin enhanced *meta*-dechlorination of PCBs, while others failed to accelerate this microbial process. They proposed that solid-state humin significantly enhanced PCB dechlorination, likely by facilitating electron transfer and improving PCB bioavailability. These findings emphasize the potential application of humic substances to improve bioremediation efficiency.

## Functional materials and synergies with OHRB

Integrating functional materials with OHRB offers exciting opportunities for enhanced pollutant degradation. Niu et al. reviewed the application of biochar and its composites for degrading chlorinated hydrocarbons. This review discussed the removal efficiency, influencing factors, and reaction mechanisms of chlorinated hydrocarbon degradation. Biochar itself can adsorb chlorinated hydrocarbons through chemical bonds on its surface, whereas biochar composite materials are able to induce the generation of free radicals to degrade chlorinated hydrocarbons. Furthermore, biochar coupled with microorganisms facilitates pollutants degradation by establishing an “electron shuttle bridge” between biological and non-biological components.

## Interplay of redox processes in complex contaminations

Bioremediation at contaminated sites is often challenged by the coexistence of diverse pollutants. Yang et al. investigated the interactions between nitrate reduction and trichloroethene (TCE) dechlorination. Their findings highlighted a complex dynamic: low TCE concentrations stimulated nitrate reduction, while nitrate inhibited TCE dechlorination to dichloroethene but enhanced the detoxification of vinyl chloride to ethene. Mechanistic insights indicate that nitrate reduction's thermodynamic advantage suppresses TCE dechlorination, while self-alkalization stabilizes pH, enhancing VC dechlorination. Notably, *Dehalococcoides*, a key TCE-degrading genus, showed tolerance to high concentrations of nitrate. These observations suggest that self-regulated pH stabilization by microbial consortia can be leveraged to optimize simultaneous remediation of co-contaminants.

## Future directions

This Research Topic underscores the importance of an integrated approach to microbial dehalogenation, combining advanced mechanistic insights with functional material innovations. While the synergistic effects of materials and microorganisms hold great promise, the complexity of real-world contaminated environments necessitates continued exploration of intertwined pollutant dynamics and tailored bioremediation solutions. Future research should focus on scaling up these insights to field applications, ensuring they address the pressing challenges of environmental pollution and contribute to the broader goal of sustainable development. Additionally, microbial dehalogenation and detoxification of other organohalides of increasing concerns, such as per- and polyfluoroalkyl substances, remains understudied, requiring focused research efforts. Theory-guided design of dehalogenating consortia and bioremediation strategies also presents a new avenue for exploration, offering the potential to optimize degradation efficiency and expand the applicability of these approaches.
